# Cognitive and Functional Impairment in Stroke Survivors with Basilar Artery Occlusive Disease

**DOI:** 10.1155/2015/971514

**Published:** 2015-06-03

**Authors:** Kenia Repiso Campanholo, Adriana Bastos Conforto, Carolina Medeiros Rimkus, Eliane Correa Miotto

**Affiliations:** ^1^Department of Neurology, University of São Paulo, 05403-000 São Paulo, SP, Brazil; ^2^Stroke Clinic, Department of Neurology, University of São Paulo, 05403-000 São Paulo, SP, Brazil; ^3^Department of Radiology, University of São Paulo, 05403-000 São Paulo, SP, Brazil

## Abstract

*Background*. Despite growing literature on posterior vascular disease, specific information about the cognitive and functional profiles of patients with basilar artery occlusion disease (BAOD) is scarce. The aims of this study were (1) to compare the cognitive statuses of BAOD survivors versus healthy controls and (2) to correlate the functional capacity outcomes with the cognitive profiles of BAOD patients. *Methods*. Comprehensive cognitive and functional assessments were carried out in 28 patients with BAOD and 27 age- and education level-matched healthy controls. *Results*. Compared to matched controls, patients presented impairments in selective, sustained, and set-shifting attention, processing speed, visuospatial skills, mental flexibility, and monitoring rules. There were significant deficits in verbal episodic memory (immediate and delayed recall) and visuospatial episodic memory (immediate and delayed recall and recognition). Functional capacity outcomes were significantly related to the cognitive test results. Seventy-five percent of patients had a Modified Rankin Scale score of 0 or 1. *Conclusions*. Our results indicate good functional outcomes in a selected group of BAOD survivors, despite the presence of subnormal performance on some cognitive tests, including tests of attention, executive function, and long-term memory.

## 1. Introduction

Among all cases of stroke, 80% are ischemic, and 25% of infarcts are located in the vertebrobasilar arterial territory [1, 2]. Reports of cognitive assessment in patients with vertebrobasilar infarcts are scarce and include patients with heterogeneous arterial lesions [3–5]. Poor performance can be detected across numerous cognitive domains, such as executive function, attention, memory [6], visuospatial function [6, 7], and language [6, 8]. In particular, patients with infarcts in the basilar artery (BA) territory can present with impairments in executive function and episodic memory [4]. These cognitive deficits have been attributed to involvement of neural networks in the brainstem, cerebellum, and upper cortex [5]. Despite these cognitive deficits, good functional outcomes have been reported in up to 78% of survivors at 6 months [9, 10] and 1 year [11] after stroke, probably due to preservation of the eloquent cerebral regions. Functionality and quality of life were favorable in approximately 75% of basilar artery occlusion disease (BAOD) survivors at 4.2 years after stroke [12].

Previous studies have not considered the relationship between neuropsychological testing outcomes and functionality scales. Until now, specific information about the cognitive and functional profiles of BAOD patients has not been known. Therefore, the aims of this preliminary study were (1) to compare the cognitive statuses of BAOD survivors versus age- and education level-matched controls and (2) to correlate functional capacity outcomes with cognitive variables for BAOD patients.

## 2. Methods

This case-control study was carried out at the Stroke Clinic of the Clinical Hospital of São Paulo University Medical School. The study protocol was approved by the Institutional Ethics Committee. All patients provided informed consent to participate.

### 2.1. Subjects

This study included 28 patients with ischemic stroke due to BAOD confirmed by computed tomography imaging or angiography, magnetic resonance (MR) imaging or angiography, and/or digital subtraction angiography. Cognitive assessment was performed at least 6 months after the stroke; at least 6 months had passed between the stroke and patient inclusion in the study. All patients had BAOD, defined as the presence of an infarct in the BA territory, and had 50% or greater stenosis or occlusion of the BA, as diagnosed at the time of infarction.

Patients were matched by age and education level with 27 controls recruited from the São Paulo community. There were no statistically significant between-group differences (mean [standard deviation]) in age (patients: 66.5 [11.3] years, controls: 65.6 [7.8] years, *p* = 0.631) or years of education (patients: 6.5 [5.1] years, controls: 6.4 [4.2] years; *p* = 0.868).

**Figure 1:**
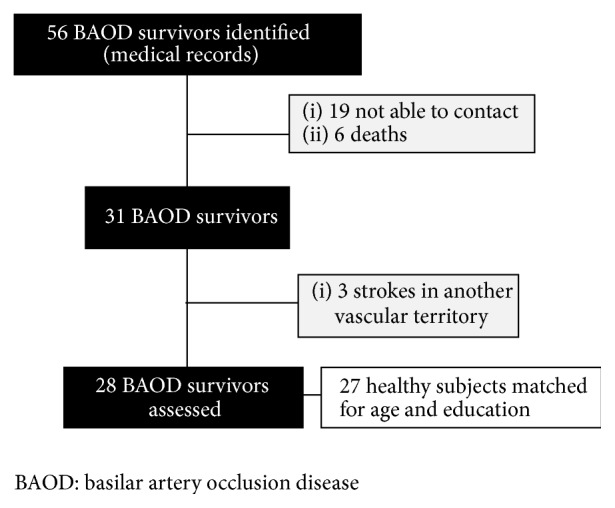
Flowchart for recruiting BAOD survivors and healthy controls.

Patients were excluded from the study if they (1) had other brain lesions such as tumors, hemorrhagic stroke, traumatic injury, epilepsy, or infectious disease, (2) had a current diagnosis of any psychiatric disorder, used alcohol or drugs, or were taking psychotropic or neuroleptic medications, (3) had a history of functional capacity impairment or complaints of cognitive deficits before the infarct that led to BAOD diagnosis, or (4) had a stroke in any other vascular territory (Figure 1). Healthy subjects were excluded if they (1) had any neurological or psychiatric disorder, (2) abused alcohol or drugs or were taking psychotropic medications, (3) had any motor, auditory, or visual disorder, (4) had lower-than-expected scores for education level on the Mini-Mental State Exam (MMSE score <20 for illiterate individuals, <25 for 1–4 years, <26.5 for 5–8 years, <28 for 9–11 years, and <29 for ≥12 years of education [13, 14]), (5) had an anxiety or depression score on the Hospital Anxiety and Depression Scale (HADS) of 9 or more [15], or (6) had a functional dependence score greater than 5 on the Pfeffer Functional Activities Questionnaire [16].

### 2.2. Clinical and Radiological Data

The following data were retrieved from medical charts and interviews with patients and their relatives: age, sex, years of education, comorbid conditions, vascular risk factors, infarct etiology, and clinical presentation. Brain and angiographic images of the patients were analyzed by an experienced neuroradiologist who was blinded to the clinical data and cognitive status of the patient. All patients had brain MR images. The neuroradiologist was asked to identify infarct location (cerebellum, brainstem, occipital lobe, thalamus, or other). BAOD was assessed by MR angiography and classified as mild (0–29% stenosis), moderate (30–69% stenosis), or severe (70–99% stenosis or occlusion). Locations of stenoses or occlusions were recorded [17, 18].

### 2.3. Neuropsychological Tests

Cognitive and functional outcomes were evaluated in a single interview by an experienced neuropsychologist. This contact occurred, on average, 6.7 years (minimum of 6 months) after the patient's first stroke. The MMSE [13, 14] and comprehensive cognitive assessment with neuropsychological tests were performed at this time. Rationale for inclusion of each test was based on previous literature showing that an extensive battery of cognitive tests demonstrates higher accuracy for identifying possible cognitive impairments in neurological patients [19].

The following neuropsychological tests were used to examine the corresponding cognitive domains: the Trail Making Test (TMT), Victoria version of the Stroop Test, Card 3 (Stroop Card 3) [20], and Symbol Digit Modalities Test (SDMT) [21] for attention and information processing speed; the Phonemic Verbal Fluency Task [22], Category Fluency Test (animals) [23], and Modified Wisconsin Card Sorting Test (MWCST) [24] for executive function; the Wechsler Adult Intelligence Scale-Digit Span [25] for short-term memory; the Hopkins Verbal Learning Test-Revised (HVLT-R) [26] and Brief Visuospatial Memory Test-Revised (BVMT-R) [27] for long-term memory; the Boston Naming Test (BNT) [28] for language; and the Visual Object and Space Perception Battery: Fragmented Letters and Position Discrimination [29] for perceptive and visuospatial ability. All cognitive abilities and tests are listed in Table 1. Mood evaluation was carried out with the HADS [15].

Functional capacities of the survivors before the first stroke and at the moment of the present study were evaluated by the Modified Rankin Scale (mRS) [30]. This scale includes seven scores that describe the functional status of patients: 0: no symptoms at all; 1: no disability despite symptoms; 2: slight disability; 3: moderate disability; 4: moderately severe disability; 5: severe disability; and 6: death.

### 2.4. Statistical Analysis

Absolute and relative frequencies as well as measures of central tendency and dispersion were used to present demographic, clinical, and radiological data. Means are followed by standard deviations in parentheses. Distributions of the data were examined by the Shapiro-Wilk normality test. Comparisons of raw scores between groups were conducted by the Mann-Whitney test for nonnormally distributed variables or by one-way ANOVA otherwise. Other comparisons were conducted with the *z*-score for cognitive variables from the appropriate normative data. When analysis of covariance was necessary, the ANCOVA test was used. Effect size was calculated with eta squared. Correlations were measured with the Spearman or Pearson correlation coefficient. A *p* value less than 0.05 was considered statistically significant.

## 3. Results

All patients had vascular risk factors, with an average of 4.6 (1.5) factors per patient. Hypertension was present in all patients. Lesions were detected in the brainstem or cerebellum in more than two-thirds of patients. The proximal BA was the most commonly affected segment by arterial stenosis or occlusion. Radiological characteristics are shown in Table 2.

Table 1 shows raw score comparisons of cognitive performance between patients and controls. There were significant differences between patients and controls in the HVLT-R and BVMT-R (immediate and delayed recall and recognition), BNT, MWCST, Position Discrimination, SDMT, TMT Part B, Stroop Card 3, and MMSE results.

Table 3 shows *z*-score comparisons of cognitive performance between patients and controls, while controlling for age and education between groups. There were significant differences between patients and controls in the HVLT-R (immediate and delayed recall), BVMT-R (immediate and delayed recall and recognition), Category Fluency Test, MWCST, Position Discrimination, SDMT, TMT Parts A and B, and Stroop Card 3 results. However, the effect sizes were small. *z*-score values for patients were negative but did not indicate severe cognitive impairment.

Six patients had relevant mood alterations or symptoms of depression, and six patients had symptoms of anxiety as assessed by the HADS. Pearson correlations indicated that anxiety symptoms were moderately and negatively related to HVLT-R recognition. Anxiety and depression had a moderate positive relationship with BVMT-R immediate recall (Table 4).

Before experiencing a stroke, none of the survivors had significant functional dependence. At the time when they were evaluated (6.7 [5.1] years after stroke), all patients had mRS scores of 3 or less; specifically, 5 patients (18%) had a score of 3, 2 patients (7%) had a score of 2, 13 patients (46%) had a score of 1, and 8 patients (29%) had a score of 0. Therefore, 75% of patients had no functional disability, and 25% had slight to moderate functional disability.

Spearman correlations between cognitive outcomes and mRS results are presented in Table 4. In univariate analyses, significant correlations were present between mRS scores and all cognitive test results, except for Digit Span, Phonemic Verbal Fluency, and Fragmented Letters. Correlation coefficients indicated a generally moderate relationship among variables, although the relationship was weak for Category Fluency and strong for SDMT.

## 4. Discussion

Compared to controls, BAOD survivors presented with impairments in selective, sustained, and set-shifting attention, processing speed, visuospatial skills, mental flexibility, and monitoring rules. There were significant deficits in verbal episodic memory (immediate and delayed recall) and visuospatial episodic memory (immediate and delayed recall and recognition). Mild to moderate correlations were found between performance on some cognitive tests and functional capacity scales.

Infarcts in the BA territory are commonly located in the thalamus, brainstem, and cerebellum. Patients with lesions in these territories, not specifically caused by BAOD, exhibit similar impairments in performance on neuropsychological testing [4, 5, 11, 31]. Patients with posterior circulation infarcts have impairments in executive function, attention, memory [6], visuospatial ability [6, 7], and language [6, 8]. These cognitive profiles have been attributed to damage in the neural networks that link anterior with brainstem and cerebellar regions [4, 5].

Encephalic regions within the BA territory are connected via the thalamus [31–33] with the parietal [34–36] and frontal cortices [4, 5]. These connections have been associated with top-down and bottom-up information processing systems related to executive monitoring, as well as attentional and perceptual systems [4, 5, 37]. It is possible that lesions in these regions (in our sample, due to BAOD) contributed to the observed cognitive test results.

Functional capacity was moderately correlated with the presence of cognitive impairment, indicating that worse functional results were due to poorer scores on cognitive tests. However, 75% of patients in this study had good mRS results, suggesting favorable functional outcomes for the BAOD survivors. In a previous study, patients with vertebrobasilar territory lesions had better functional outcomes on the mRS compared to patients with lesions in the internal carotid artery territory [38]. Another study reported a good functional prognosis at the 1-year follow-up [11], which is in line with our findings.

Our results can be explained by the small patient *z*-score values for outcomes other than SDMT, Position Discrimination, and TMT B. This finding indicates that cognitive deficits were not important, despite the statistically significant differences compared to controls. This discrepancy could be due to the small effect size.

Symptoms of anxiety and depression are frequently reported after cerebrovascular disease [39]. Even in the absence of neurological deficits, their presence is predominantly related to memory and attention deficits [40, 41]. However, in our group of patients, the frequency of anxiety or depression symptoms was relatively low. Anxiety symptoms showed a negative relationship with one long-term verbal memory variable, and the anxiety and depression symptoms were positively related to one long-term visuospatial memory variable. These conflicting results could be due to the exclusion of patients with a history of psychiatric disorders or who were currently using psychotropic medications. The low number of patients with such symptoms may have led to a floor effect for correlations with cognitive impairments.

A limitation of the present study was the relatively small sample size, which should be rectified by future investigations. Nevertheless, to our knowledge, this is the first study to investigate the long-term cognitive profiles of BAOD survivors.

## 5. Conclusion

Our sample of BAOD survivors showed impairments in episodic memory, visuospatial skills, executive function, and attention (mental flexibility, monitoring rules, and processing speed, as well as selective, sustained, and set-shifting attention). Functional capacity outcomes were significantly related to the cognitive test results. Overall, our results indicate good functional outcomes in a selected group of BAOD survivors, despite the presence of subnormal performance on some cognitive tests.

## Figures and Tables

**Table 1:** Comparison of cognitive outcomes (raw score) between BAOD patients and healthy controls.

Cognitive functions	Cognitive tests	Patients	Controls	*p*
		Mean (SD)	Mean (SD)	
Memory				
Short-term memory	Digit Span	10.0 (2.52)	10.85 (2.73)	0.208
Long-term memory (verbal episodic memory)	HVLT-R immediate recall	18.04 (5.10)	21.85 (3.11)	**0.002^*∗∗*^**
	HVLT-R delayed recall	5.04 (2.85)	6.81 (1.78)	**0.008^*∗∗*^**
	HVLT-R recognition	8.86 (2.22)	10.00 (1.11)	**0.048^*∗*^**
Long-term memory (visuospatial episodic memory)	BVMT-R immediate recall	14.78 (10.11)	19.59 (6.52)	**0.043^*∗*^**
	BVMT-R delayed recall	5.59 (4.05)	7.67 (2.90)	**0.035^*∗*^**
	BVMT-R recognition	4.85 (1.35)	5.70 (0.54)	**0.015^*∗*^**

Language				
Naming	BNT	43.89 (8.77)	48.48 (5.21)	**0.048^*∗*^**

Executive function				
Monitoring rules	Phonemic Verbal Fluency	23.73 (10.67)	26.62 (8.09)	0.277
	Category Fluency	12.11 (4.43)	13.59 (2.62)	0.095
Mental flexibility	MWCST	3.29 (1.8)	4.48 (1.50)	**0.013^*∗*^**

Perceptive and visuospatial ability				
Perceptive ability	Fragmented Letters	18.52 (2.08)	19.15 (0.93)	0.645
Visuospatial ability	Position Discrimination	18.14 (1.94)	19.59 (0.75)	**0.002^*∗∗*^**

Attention and processing speed				
Processing speed	SDMT	18.14 (12.10)	28.26 (10.57)	**0.001^*∗∗*^**
Sustained attention	TMT Part A	70.61 (55.26)	50.85 (20.28)	0.152
Set-shifting attention	TMT Part B	202.13 (86.95)	135.27 (48.08)	**0.013^*∗*^**
Selective attention	Stroop C	49.00 (22.34)	33.58 (7.29)	**0.001^*∗∗*^**

Cognitive screening	MMSE	25.09 (3.68)	27.44 (1.45)	**0.018^*∗*^**

*Note*. *∗* indicates *p* < 0.05 and *∗∗* indicate *p* < 0.01. Digit Span: number of correct responses of forward plus backward order; HVLT-R and BVMT-R: immediate recall (sum of 3 immediate recall trials), delayed recall (sum of words or figures with form and location correct), and recognition (correct hits minus false-positive responses); BNT: hits without prompts plus hits after semantic prompts; MWCST: total number of categories; Phonemic Verbal Fluency Task: sum of words beginning with F + A + S; Category Fluency Test: sum of animal names; Fragmented Letters and Position Discrimination: sum of correct responses; SDMT: sum of correct responses; TMT Parts A and B: time in seconds; Stoop C: time in seconds; and MMSE: sum of correct responses.

**Table 2:** Clinical characteristics of BAOD survivors.

Risk Factors	*N* (28)	%
Hypertension	28	100%
Dyslipidemia	22	79%
Transient ischemic attack	13	46%
Diabetes mellitus	13	46%
Smoking	12	43%
Obesity	6	21%
Heart disease	10	36%
Prior stroke	10	36%
Alcoholism	6	21%
Migraine	4	14%
Family history of stroke	5	18%
Contraceptive use	1	4%

Lesion location	*N* (28)	%

Cerebellum	20	71%
Brainstem	19	68%
Occipital	11	39%
Thalamus	10	36%

Stenosis location	*N* (28)	%

Proximal	20	71%
Medial	11	39%
Distal	8	29%
Multiple	8	29%

Severity of stenosis	*N* (28)	%

Mild	7	25%
Moderate	8	29%
Severe	10	36%
Occlusion	3	11%

Mood	*N* (28)	%

Depression	6	22%
Anxiety	6	22%

**Table 3:** Comparison of cognitive outcomes (*z*-scores) between BAOD patients and healthy controls.

Cognitive tests	Patients	Controls	*p*	Effect size
	Mean (SD)	Mean (SD)		
Digit Span	−0.10 (0.82)	0.06 (0.68)	0.417	0.013
HVLT-R immediate recall	−0.47 (0.88)	0.19 (0.61)	**0.002** ^*∗∗*^	**0.178**
HVLT-R delayed recall	−0.91 (1.12)	−0.09 (0.76)	**0.003** ^*∗∗*^	**0.159**
HVLT-R recognition	−0.58 (1.38)	−0.03 (0.72)	0.055	0.070
BVMT-R immediate recall	−0.29 (1.25)	0.39 (0.71)	**0.018** ^*∗*^	**0.106**
BVMT-R delayed recall	−0.47 (1.18)	0.25 (0.78)	**0.012** ^*∗*^	**0.117**
BVMT-R recognition	−0.33 (1.28)	0.43 (0.47)	**0.005** ^*∗∗*^	**0.146**

BNT	0.61 (1.21)	1.07 (0.79)	0.061	0.067

Phonemic Verbal Fluency	−0.46 (1.07)	−0.12 (0.91)	0.227	0.030
Category Fluency	−0.53 (0.97)	−0.02 (0.73)	**0.036** ^*∗*^	**0.083**
MWCST	−1.01 (1.17)	−0.18 (0.84)	**0.002** ^*∗∗*^	**0.181**

Fragmented Letters	−0.90 (2.08)	−0.25 (0.92)	0.137	0.046
Position Discrimination	−1.93 (2.42)	−0.13 (0.94)	**0.001** ^*∗∗*^	**0.198**

SDMT	−2.24 (1.24)	−1.21 (1.11)	**0.001** ^*∗∗*^	**0.204**
TMT Part A	−1.27 (2.79)	0.25 (1.38)	**0.015** ^*∗*^	**0.120**
TMT Part B	−1.76 (2.36)	−0.08 (1.00)	**0.002** ^*∗∗*^	**0.202**
Stroop C	−0.66 (1.76)	0.68 (0.72)	**0.001** ^*∗∗*^	**0.212**

*Note*. *∗* indicates *p* < 0.05 and *∗∗* indicate *p* < 0.01. Controls were matched with patients for age and education level.

**Table 4:** Correlations among cognitive variables (*z*-scores), anxiety and depression symptoms, and functional capacity variables.

Cognitive tests	mRS		Anxiety symptoms		Depression symptoms	
	*r*	*p*	*r*	*p*	*r*	*p*
Digit Span	−0.222	0.103	−0.210	0.292	−0.037	0.854
HVLT-R immediate recall	−0.526^*∗∗*^	<0.001	−0.194	0.333	−0.136	0.498
HVLT-R delayed recall	−0.467^*∗∗*^	<0.001	0.037	0.855	0.089	0.659
HVLT-R recognition	−0.476^*∗∗*^	<0.001	−0.410^*∗*^	0.034	−0.282	0.153
BVMT-R immediate recall	−0.542^*∗∗*^	<0.001	0.399^*∗*^	0.044	0.527^*∗∗*^	0.006
BVMT-R delayed recall	−0.524^*∗∗*^	<0.001	0.148	0.462	0.370	0.058
BVMT-R recognition	−0.465^*∗∗*^	<0.001	0.012	0.953	0.242	0.233
BNT	−0.423^*∗∗*^	0.001	−0.124	0.538	0.096	0.634
Phonemic Verbal Fluency	−0.237	0.091	−0.272	0.189	0.011	0.960
Category Fluency	−0.325^*∗*^	0.016	−0.081	0.689	0.048	0.810
MWCST	−0.565^*∗∗*^	<0.001	0.172	0.392	0.223	0.263
Fragmented Letters	−0.067	0.640	−0.421^*∗*^	0.040	−0.296	0.161
Position Discrimination	−0.570^*∗∗*^	<0.001	−0.012	0.952	−0.002	0.993
SDMT	−0.679^*∗∗*^	<0.001	−0.063	0.755	0.093	0.644
TMT Part A	0.598^*∗∗*^	<0.001	0.138	0.521	0.150	0.484
TMT Part B	0.599^*∗∗*^	<0.001	0.114	0.603	0.241	0.269
Stroop Card 3	0.568^*∗∗*^	<0.001	0.106	0.631	0.012	0.956

*Note*. *∗* indicates *p* < 0.05 and *∗∗* indicate *p* < 0.01.
